# The effect of perceived professional benefits on health professionals’ job engagement: the role of psychological availability and future perceived professional benefits

**DOI:** 10.1186/s12913-024-10684-y

**Published:** 2024-02-21

**Authors:** Jin Wan, Wenjun Zhou, Mingyue Qin, Haiming Zhou

**Affiliations:** 1 East China Jiaotong University, 330013 Nanchang, China; 2 Research centre for high speed railway and regional development, East China Jiaotong University, 330013 Nanchang, China; 3 Jiangxi institute of talent and industry integration development, East China Jiaotong University, 330013 Nanchang, China; 4 Shandong University of Science and Technology, 271000 Taian, China

**Keywords:** Perceived professional benefits, Psychological availability, Future perceived professional benefits, Job engagement, Moderated mediation model

## Abstract

**Background:**

Improving the job engagement of health professionals can effectively enhance the quality of their medical services. However, few studies have investigated whether and how perceived professional benefits affect job engagement. Based on resource conservation theory, this study explored the effect of the influence of perceived professional benefits on job engagement, and also examined the mediating role of psychological availability and the moderating role of future perceived professional benefits.

**Methods:**

A cross-sectional study was conducted in six tertiary hospitals and seven secondary hospitals in Liu Panshui, a city in western China. A total of 1,406 valid questionnaires were obtained and analysed by using correlation analysis, hierarchical regression analysis, and bootstrap tests.

**Result:**

The study found a significant positive association between health professionals’ perceived professional benefits and their job engagement. Additionally, psychological availability was found to mediate this relationship. Future perceived professional benefits not only positively moderate this relationship between perceived professional benefits on health professionals’ psychological availability but also positively moderate the mediating role of psychological availability between perceived professional benefits and job engagement.

**Conclusion:**

Improving health professionals’ perceived professional benefits can enhance their job engagement by increasing their psychological availability. However, for health professionals with low future perceived professional benefits, this improvement may disappear. Therefore, it is important to enhance both their current and future perceived professional benefits to improve their job engagement.

**Supplementary Information:**

The online version contains supplementary material available at 10.1186/s12913-024-10684-y.

## Background

Health professionals are encountering challenges such as heightened work pressure and extended working hours, which can lead to burnout and hinder their full engagement in work [1]. Previous studies have shown that health professionals’ job engagement can foster the development of organizational citizenship behaviour [2], significantly enhance their job performance [3] and mental health [4, 5], and reduce their job burnout and turnover intention [6, 7]. Job engagement is a positive state in which a health professionals are energetic at work and less likely to compromise their professionalism in the face of difficulties; so fully engaged that they are not easily distracted by external influences, and so passionate that they are willing to give it their all [8]. Improving health professionals’ job engagement can effectively enhance their work status and the quality of medical services.

Previous studies have shown that emotional intelligence, self-efficacy, organizational support, and relational coordination have a significant positive impact on the job engagement of health professionals [9–12]. However, psychological stress, occupational stress and stressful events can negatively predict job engagement [13–15]. Self-efficacy and perceived organizational support are subjective experiences generated by individuals based on their experiences. According to expectation theory, individuals hold expectations at work, such as the expectation of being recognized and treated fairly by leaders [16]. Fulfillment of employees’ expectations means they have achieved their desired goals, which can improve their self-efficacy. It also indicates that the organization or significant others within the organization have provided the necessary recognition and support, leading to a sense of organizational support among employees [17]. Therefore, health professionals’ perceptions of professional benefits improve their job engagement by promoting their self-efficacy and perceived organizational support. Perceived professional benefits refer to employees’ perceptions of the gains and benefits from their job [18]. Research indicates that there is significant room for improvement in health professionals’ perceived professional benefits [19]. Perceived professional benefits among health professionals not only enhance their professional identity and job satisfaction but also have a significant positive impact on retention [18, 20]. Job satisfaction and professional identity are both key factors affecting health professionals’ job engagement [21, 22]. However, most of the existing studies focus on the antecedents of perceived professional benefits [18, 19, 23, 24], and few examining the relationship between perceived professional benefits and job engagement. Therefore, the first purpose of this study is to explore the effects of perceived professional benefits on job engagement.

Resources refer to the things that an individual considers valuable, including material, condition, individual characteristics, and energy [25].. Different dimensions of perceived professional benefits belong to different types of resources. According to resource conservation theory, perceived professional benefits as a resource [18], can reduce the resource loss caused by work requirements and improve psychological availability. Psychological availability refers to an individual’s physical, emotional or psychological resources at a given moment. The higher the psychological availability, the greater the individual’s perceived resources [26]. As a positive emotional experience in nursing practice, enhancing the health professional’s perceived professional benefits can effectively improve the perception of individual resources. Resource conservation theory also emphasises that individuals with greater resources are less vulnerable to resource loss and more capable of acquiring new resources [25]. Therefore, they are more likely to devote themselves to work, accept work challenges and experience lower burnout and higher job engagement [27, 28]. Studies have indicated that psychological availability has a significant positive effect on job engagement [29–31]. Therefore, the second purpose of this study is to find the relationships among perceived professional benefits, psychological availability and job engagement.

The resources individuals believe they have are affected not only by the current perceived professional benefits but also by the future perceived professional benefits. Future perceived professional benefits are the expectation of future benefits that will promote development [32]. Resource conservation theory suggests that individuals tend to preserve, protect, and acquire resources [25], and both the potential threat of resource loss and the actual loss of resources will trigger tension and stress [33]. Therefore, individuals with low future perceived professional benefits represent a potential threat of resource loss relative to the present. This threat will cause tension and stress in individuals, and under tension and pressure, their attention and focus are more likely to be attracted by negative factors, while ignoring or reducing their attention to positive factors [34]. This leads to a decrease in the positive experience they have with their current job, the positive effect of perceived professional benefits on psychological availability is reduced. On the contrary, when future perceived professional benefits is high, the current perceived professional benefits has a stronger positive effect on psychological availability. Therefore, the third purpose of this study is to explore future perceived professional benefits moderating effect.

**Fig. 1:**
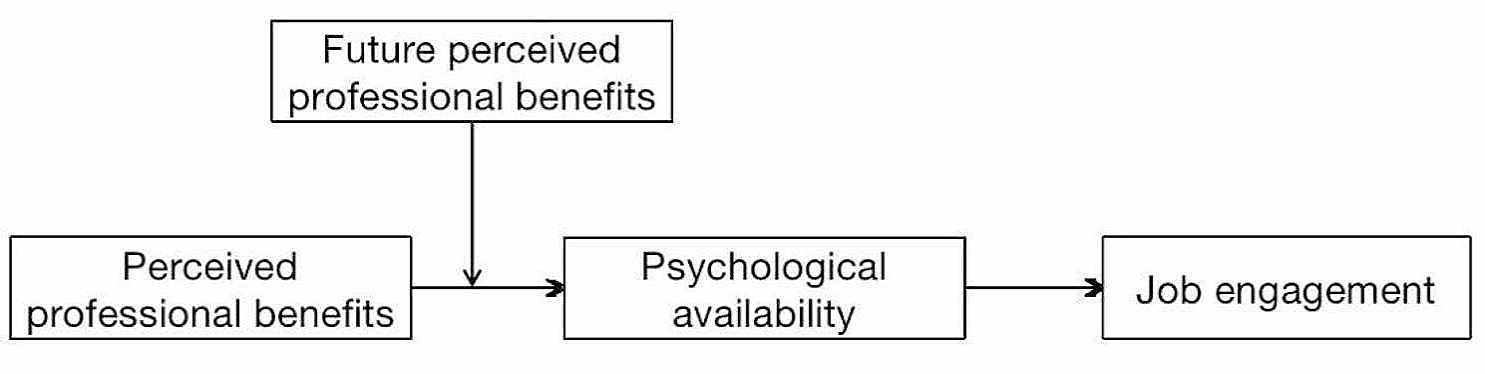
The research model

Therefore, this study developed a moderated mediation model (Fig. 1). Based on resource conservation theory, we examined the mediating effect of psychological availability and the moderating effect of future perceived professional benefits. Our findings provide practical implications for enhancing the job engagement of health professionals.

## Methods

### Design

A cross-sectional design was used in this study.

### Sample

Stratified random sampling was conducted in secondary and tertiary hospitals in Liu Panshui, a city in western China. A two-stage sampling was performed, using clusters (hospitals) in the first phase and stratums (occupational groups) in the second phase. The survey hospitals were selected from each type of hospital by assigning a number to each hospital and using the lottery method. A total of six tertiary hospitals and seven secondary hospitals were selected. In each hospital, respondents were randomly sampled using the lottery method for each occupation based on their proportion of staff. Only those who had been working there for more than three months were included in the survey. After explaining the survey’s purpose and ensuring confidentiality, we obtained consent and distributed paper questionnaires at their offices.

The number of parameters to be estimated in the research model was calculated, including factor loadings, path coefficients, and residual coefficients. Based on the requirement of 10 samples for each parameter, this study has a total of 47 estimated parameters. Therefore, the sample size should be more than 470. Based on the standard recovery rate of 50% and the 50% efficiency of the recovered questionnaires, 2,000 questionnaires were initially distributed. Out of these, 1,679 were returned, and after excluding invalid questionnaires (i.e. those with the same option chosen for more than 90% of the items, less than 90% complete, and with variable scores exceeding 3 standard deviations), 1406 valid questionnaires were obtained.

### Assessment tools

#### Perceived professional benefits scale

Perceived professional benefits refer to employees’ perceptions of the gains and benefits from their job [18]. According to Maslow’s hierarchy of needs theory, perceived professional benefits were compiled from economic, social, respect and development dimensions [35]. The scale includes five items, such as “I perceive that I have gained the income I deserve”. A five-point Likert scale was used, and the responses ranged from 1 (strongly disagree) to 5 (strongly agree). A higher score implied stronger perceived professional benefits. The Cronbach’s α coefficient in this study was 0.77.

#### Psychological availability scale

Psychological availability refers to an individual’s physical, emotional or psychological resources at a certain moment [26]. The psychological availability scale was developed in May 2004 [29]. The scale includes five items, such as “I am confident in my ability to handle competing demands at work”. A five -point Likert scale was used, and the responses ranged from 1 (strongly disagree) to 5 (strongly agree). A higher score implied stronger psychological availability. The Cronbach’s α coefficient in this study was 0.83.

#### Future perceived professional benefits scale

Future perceived professional benefits refer to employees’expectation of future gains and benefits from their job. Future perceived professional benefits are consistent with the measurement of perceived professional benefits. But these questions don’t ask about current perceived professional benefits, they ask about his/her estimate of future perceived professional benefits. The Cronbach’s α coefficient in this study was 0.93.

#### Job engagement scale

Job engagement is a positive state of work that reflects the degree of energy, dedication, and focus shown in the work [8]. The job engagement scale was developed by Saks in 2006 [36]. The scale includes five items, such as “Sometimes I am so into my job that I lose track of time”. A five-point Likert scale was used, and the responses ranged from 1 (strongly disagree) to 5 (strongly agree). A higher score implies stronger job engagement. The Cronbach’s α coefficient in this study was 0.70.

### Control variables and marker variable

It was found that gender, age, education, and length of service in the organization can affect the job engagement of health professionals [37], so this study took them as the control variables.

In this study, the marked-variable method was used to test for common method bias, which means that the significance of the correlation coefficients between the variables did not change significantly after controlling for the marked variables, indicating that there is no serious common method bias [38]. Psychological detachment was used as a marker variable that had no theoretical relationship with this study. Psychological detachment is a state of mind in which an individual detach from work tasks during non-work hours. The psychological detachment scale was developed by Sonnentag & Fritz (2007) [39]. The scale includes four items, such as “After work, I forget about work”. A five-point Likert scale was used, and the responses ranged from 1 (strongly disagree) to 5 (strongly agree). A higher score implies stronger psychological detachment. The Cronbach’s α coefficient in this study was 0.73.

### Data analysis

SPSS 25.0 was used for the reliability test, common method bias test, correlation analysis and hierarchical regression analysis to test the reliability of the scales, the effect of perceived professional benefits on job engagement, the mediating effect of psychological availability, the moderating effect of future perceived professional benefits between perceived professional benefits and psychological availability. The p value smaller than 0.05 considered a statistically significant difference. Bootstrapping was used to test the moderated mediation model, and the parameter estimation was performed using the great likelihood method. The test level was set at α = 0.05, and if the 95% CI of the standardized path coefficients did not contain 0, the effect was significant.

## Results

### Description of respondents

The demographic and clinical characteristics of health professionals are presented in Table 1. Among respondents, 68.8% were female and 31.2% were male; 43.5% were nurses, 35.7% were doctors and others were pharmacists and administrative staff; 42.5% had a college degree or less, 56.7% had a bachelor’s degree; the average age was 32.7 years old, with a standard deviation of 7.83. According to the 2020 China Health Statistics Yearbook, which was published in 2021, coinciding with our data collection. 74.4% health professionals were female and 25.6% were male. 49.0% with bachelor degree or above; 51.0% were under the age of 35, and 48.3% had worked for less than 10 years. This means that the characteristics of the final sample are similar to the same characteristics in the population.

**Table 1:** Demographic and clinical characteristics of the sample(*N* = 1406)

Variable	Cluster	N(%)
Gender	Male	439(31.2%)
	Female	967(68.8%) 0)
Age	≦ 30 years	671(47.7%)
	≧ 31vyears 4	735(52.3%)
Marriage	Married	415(29.5%)
	Unmarried	954(67.9%)
	Divorced or widowed	37(2.6%)
Education	College degree or less	597(42.5%)
	Bachelor’s degree	797(56.7%)
	Master’s degree or above	12(0.8%)
Position	Nurse	612(43.5%)
	Doctor	502(35.7%) 9 9
	Pharmacist	194(13.8%)
	Administrative staff	98(7.0%)
Working years	≦ 10 years	884(62.9%)
	≧ 10 years 4	522(37.1%)

### Discriminant validity analysis

Confirmatory factor analysis was performed on all variables in the research model using Mplus7.0 to test the discriminant validity between variables. This study compared the fit of models with one to four factors. The fitting index of the four-factor model (χ2 = 965.347, df = 146,χ2/df = 6.612, CFI = 0.936, TLI = 0.925, SRMR = 0.065, RMSEA = 0.063) was significantly better than that of the other models. Specifically, the factor loading ranges of perceived professional benefits, psychological availability, future perceived professional benefits, and job engagement were 0.636–0.810, 0.566–0.812, 0.574–0.831, and 0.776–0.894, respectively. All parameters met statistical criteria, indicating that the five research variables had good discriminant validity.

### Common method bias test

Considering that all the data were self-reported by the research subjects at one time point, the following analysis was conducted to reduce the impact of common method bias. According to Scott Vrieze’s suggestion, the comparative Bayesian information criterion (BIC) was used to compare models with and without common method bias. When ΔBIC is greater than 10, the model with a smaller BIC is better than the model with a larger BIC. Psychological detachment was used as a marker variable that had no theoretical relationship with this study. The model after adding the marker variable was compared with the research model (ΔBIC = 200.13, ΔBIC > 10), and the research model was significantly better than the model with a marker variable, indicating that there was no significant common method bias.

### Descriptive statistical results

The mean, standard deviation, and correlation coefficients of the variables are shown in Table 2. Gender, age, marriage, education, position, and working years were significantly correlated with the research variables, so they were treated as control variables. The results showed that perceived professional benefits are positively correlated with job engagement (*r* = 0.077, *p* < 0.01) and positively correlated with psychological availability (*r* = 0.252, *p* < 0.01), and psychological availability is positively correlated with job engagement (*r* = 0.406, *p* < 0.01).

**Table 2:** Pearson’s correlation coefficients between study variables

Variables	1	2	3	4	5	6	7	8	9	10
1.Gender										
2.Age	0.064^*^									
3.Marriage	-0.021	0.555^**^								
4.Education	0.168^**^	0.058^*^	0.078^**^							
5.Position	0.323^**^	0.183^**^	0.021	0.133^**^						
6.Working years	0.001	0.834^**^	0.482^**^	0.014	0.110^**^					
7.Perceived professional benefits	0.043	-0.060^*^	-0.095^**^	-0.165^**^	0.047	-0.053^*^				
8.Psychological availability	-0.089^**^	0.081^**^	0.023	-0.066^*^	-0.005	0.103^**^	0.252^**^			
9.Future perceived professional benefits	-0.050	0.072^**^	0.016	-0.095^**^	-0.007	0.097^**^	0.467^**^	0.368^**^		
10.Job engagement	-0.100^**^	0.089^**^	0.011	-0.041	-0.055^*^	0.136^**^	0.077^**^	0.406^**^	0.268^**^	

Note: *N* = 1406; * Correlation significant at the 0.05 level (two-tailed). ** Correlation significant at the 0.01 level (two-tailed). *** Correlation significant at the 0.001 level (two-tailed)

### Hierarchical regression analysis

As shown in Table 3, the relationship between variables was tested using hierarchical regression analysis, the F value of each model is significant. Four conditions must be met to establish the model assumed in this study: (1) The positive effect of perceived professional benefits on job engagement is significant; (2) Perceived professional benefits has a significant positive effect on psychological availability; (3) Psychological availability has a significant positive effect on job engagement; (4) The interaction term between perceived professional benefits and future perceived professional benefits has a significant effect on psychological availability. To avoid the collinearity problem, the perceived professional benefits and the future perceived professional benefits were centralized before constructing the interaction term. Gender, age, marriage, education, position, and working years were treated as control variables. In M1 and M4, only the control variables are added to the regression equation. Based on M1 and M4, Women’s psychological availability and job engagement levels were significantly higher than men’s (*β*=-0.087, *p* < 0.01; *β*=-0.083, *p* < 0.01); As working years increase, the level of employee job engagement decreases (*β*=-0.088, *p* < 0.01).

**Table 3:** Hierarchical regression analysis

Control variables	Psychological availability			Job engagement		
	M1	M2	M3	M4	M5	M6
Gender	-0.087^**^	-0.101^***^	-0.076^**^	-0.083^**^	-0.088^**^	-0.049
Age	0.022	0.028	0.034	-0.010	-0.008	-0.019
Marriage	-0.034	-0.016	-0.014	-0.097^*^	-0.064^*^	-0.058^*^
Education	-0.060^*^	-0.021	-0.013	-0.040	-0.025	-0.017
Position	0.017	0.003	0.016	-0.036	-0.041	-0.043
Working years	-0.019	-0.048	-0.086^**^	-0.088^**^	-0.098^***^	-0.079^**^
Independent variable						
Perceived professional benefits		0.265^***^	0.106^***^		0.096^***^	-0.007
Mediating variable						
Psychological availability						0.390^***^
Moderating variable						
Future perceived professional benefits			0.340^***^			
Interaction term						
Perceived professional benefits*Future perceived professional benefits			0.134^***^			
R^2^	0.023	0.089	0.178	0.042	0.051	0.189
F	4.691^***^	17.133^***^	30.248^***^	8.768^***^	9.336^***^	36.199^***^
△R^2^	0.023	0.066	0.089	0.042	0.009	0.138

Note: *N* = 1406; *p < 0.05, **p < 0.01, ***p < 0.001, two-sided test

To explore the relationship between perceived professional benefits and job engagement, based on M4, perceived professional benefits were added to obtain M5. In M5, perceived professional benefits promote job engagement (*β* = 0.096, *p* < 0.001).

To explore the relationship between perceived professional benefits and psychological availability, based on M1, perceived professional benefits were added to obtain M2. In M2, perceived professional benefits had a significant positive effect on psychological availability (*β* = 0.265, *p* < 0.001). To explore the relationship between psychological availability and job engagement, based on M5, psychological availability was added to obtain M6. In M6, psychological availability had a positive effect on job engagement (*β* = 0.390, *p* < 0.001), and the impact of perceived professional benefits on job engagement was no longer significant (*β*=-0.007, *p* > 0.05), indicating that psychological availability played a fully mediating role between perceived professional benefits and job engagement.

**Fig. 2:**
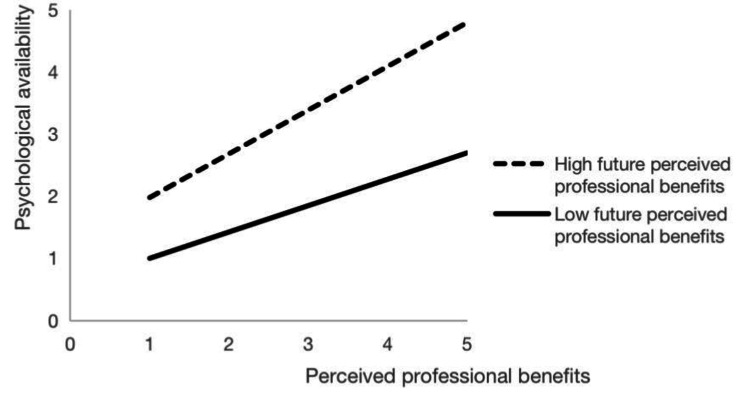
The effect of perceived professional benefits on psychological availability

To explore the moderating effect of future perceived professional benefits, the interaction term of perceived professional benefits and future perceived professional benefits was constructed. based on M2, the interaction term was added to obtain M3. In M3, the interaction term significantly positively affected psychological availability (*β* = 0.134, *p* < 0.001), indicating that future perceived professional benefits had a significant moderating effect on the relationship between perceived professional benefits and psychological availability. A simple slope analysis method was used to analyze the moderating effect of future perceived professional benefits. Taking the mean of future perceived professional benefits plus or minus one standard deviation as the high group and low group, the influence of perceived professional benefits on psychological availability in the high and low future perceived professional benefits groups was tested. The moderating effect is shown in Fig. 2.

### Moderated mediation test

Model 7 was validated by using the Process plug-in in SPSS25.0. The results of bootstrap sampling analysis for 2000 times showed that the index of moderated-mediation was 0.039, 95%CI =[0.021, 0.059]. Specifically, the positive effect of perceived professional benefits on psychological availability was significant, *β* = 0.090, CI=[0.043, 0.137]. Psychological availability had a significant positive effect on job engagement, *β* = 0.386, CI=[0.337, 0.436]. The indirect effect of perceived professional benefits on job engagement was significant through psychological availability, *β* = 0.035, CI=[0.014, 0.058]. In addition, as shown in Table 4, for health professionals with low future perceived professional benefits, the effect of perceived professional benefits on job engagement through psychological availability was not significant, *β* = 0.001, CI=[-0.029, 0.032]. For health professionals with medium and high future perceived professional benefits, perceived professional benefits had a positive effect on job engagement through psychological availability, and the influence gradually increased with the improvement of future perceived professional benefits. In conclusion, future perceived professional benefits can positively moderate the mediating effect of psychological availability between perceived professional benefits and job engagement of health professionals.

**Table 4:** The impact of perceived professional benefits on job engagement through psychological availability under different levels of future perceived professional benefits

Future perceived professional benefits	Effect	BootSE	LLCI	ULCI
Low	0.001	0.015	-0.029	0.032
Medium	0.035	0.011	0.014	0.058
High	0.068	0.013	0.044	0.094

Note: The value of low future perceived professional benefits is -SD, and the high value is + SD. LLCI is the lower bound of the confidence interval, and ULCI is the upper bound of the confidence interval

## Discussion

Based on resource conservation theory, this study found that perceived professional benefits enhance health professionals’ job engagement through psychological availability, and future perceived professional benefits play a positively moderating role in the model. For health professionals with low future perceived professional benefits, perceived professional benefits do not have a significant impact on their job engagement through psychological availability.

Specifically, first, this study found that individuals with higher perceived professional benefits were more willing to actively engage in work, which was consistent with the results of previous studies [18]. This enriches the study of the consequences of the role of perceived professional benefits, perceived professional benefits as a work motivator, has a significant predictive effect on individual work enthusiasm and job satisfaction [40]. At the same time, this is in line with the expectation of social exchange theory. When individuals perceive professional benefits, they are more likely to provide positive feedback and take initiative to improve their job engagement.

Second, this study found that perceived professional benefits indirectly promoted the job engagement of health professionals through psychological availability. As the sum of the sense of identity, sense of honor, sense of accomplishment, material and spiritual generated from work, perceived professional benefits constitute the psychological resources of individual work. Therefore, individuals with higher perceived professional benefits are perceived to have more resources. Resource conservation theory points out that an individual’s resource reserves can improve emotional exhaustion and fatigue caused by resource consumption due to work requirements, and they will have higher enthusiasm and a higher level of job engagement [41]. This study confirms that perceived professional benefits can indeed repair the resources consumed due to job requirements, improve the availability of psychological resources, and thus more actively engage in work. This conclusion expands the research on the influence mechanism of perceived professional benefits on job engagement.

Finally, future perceived professional benefits not only positively moderate the effect of perceived professional benefits on the psychological availability of health professionals but also positively moderate the mediating effect of psychological availability on the relationship between perceived professional benefits and job engagement. For health professionals with high future perceived professional benefits, perceived professional benefits have a greater impact on their job engagement through psychological availability. However, for health professionals with low future perceived professional benefits, the effect of perceived professional benefits on job engagement through psychological availability was not significant. Based on resource conservation theory, individuals may perceive a potential threat of resource loss and experience tension and stress when they anticipate low levels of future professional benefits, leading to a reduction in psychological availability. The study findings demonstrate the boundary conditions of the indirect impact of perceived professional benefits on the job engagement of health professionals. The study also establishes a link between current and future perceived professional benefits and proposes a new approach to enhance their psychological availability and job engagement.

### Implications for practice

Based on the findings of this study, it is possible to indicate some implications for future clinical practice and policy. First, hospital managers should optimize the management system to create a healthy, equal and humanized working environment for health professionals. At the same time, we should implement the matching salary and welfare system and pay attention to the effective incentive for health professionals. Furthermore, it is important to provide regular professional skills training to enhance the quality of health professionals. This will enable them to gain the respect and trust of patients through their solid theoretical knowledge and technical expertise. Second, hospital managers should help health professionals quickly recover individual self-regulation resources so that they can face various pressures and setbacks at work with positive and optimistic attitudes. At the same time, to ensure that health professionals can better complete their work, hospital management should provide them with relevant work resources. This will improve their psychological availability due to the perception of work-related resources, and they will exhibit a higher level of job engagement. Furthermore, when organising work, the hospital should also fully consider each individual’s abilities, assist them in meeting the demanding job requirements, and improve their psychological availability so that they can be more engaged. Finally, hospitals should establish a steady increase in salary mechanisms, establish a reasonable promotion mechanism, and open up career development paths so that health professionals can build confidence in future career development, generate high future perceived professional benefits, and improve their level of job engagement.

### Limitations

Among the limitations that the study may pose are those derived from the methodology. First, the variables in this study were all self-reported by the participants at one time point. Although statistical tests indicated no significant common method bias, future research should invite supervisors to evaluate the job engagement of their subordinates. Second, this study was a cross-sectional study, which does not adequately reflect the causal relationship between variables. A longitudinal study design should be adopted to verify the causal relationship between variables. Finally, the effect of perceived professional benefits on job engagement among health professionals may also be moderated by other individual factors, such as goal orientation. Therefore, subsequent research can further investigate the boundary conditions of perceived professional benefits’ impact on health professionals’ job engagement.

### Statement

The research design adopts questionnaire survey. Questionnaires follow the principle of voluntary participation,and the process will not cause any harm to the subjects. Before the start of the formal investigation,the subjects were informed in detail about the purpose of the research,and the procedures were formal and legal. All data collected is strictly confidential and used for academic research only,and there is no conflict of interest in research content and research results.

### Electronic supplementary material

Below is the link to the electronic supplementary material.

Supplementary Material 1

## Data Availability

The raw data supporting the conclusions of this article will be made available by the authors, without undue reservation. If someone wants to request the data from this study they can be contacted at 734141500@qq.com.

## Declarations

All methods were performed in accordance with the declaration of Helsinki.
