# Supplementary material for: The effect of perceived professional benefits on health professionals’ job engagement: the role of psychological availability and future perceived professional benefits
Source: BMC Health Serv Res. 2024 Feb 21;24:227. doi: 10.1186/s12913-024-10684-y (PMC10882821; doi:10.1186/s12913-024-10684-y)
Supplement: Supplementary file 1 — Supplementary Material 1 [file 12913_2024_10684_MOESM1_ESM.docx]

**Health professionals work status questionnaire**

This survey aims at the status of health professionals’ perceived professional benefits to inform management improvement. It will take about 10 minutes of your valuable time. There is no right or wrong answer, just answer according to your true feelings. We guarantee strict confidentiality, the report only presents the overall situation of the organization and does not involve your personal information. The credibility of the research results depends on your serious answers. Please read it carefully and fill in the answers truthfully. Thank you for your participation!

**Your background information:**

1. Gender: (1) Female (2) Male

2. Age:

3. Marriage: (1) Unmarried (2) Married (3) Divorced or widowed

4. Education: (1)College degree or less (2)Bachelor's degree (3)Master's degree or above

5. Position:(1)Nurse (2)Doctor (3)Pharmacist (4)Administrative staff

6. Working years:

**Part A Perceived professional benefits scale**

| Below is a description of your workplace and professional status, please select the option that best matches your actual situation (in general) by ticking the appropriate number. | Strongly disagree | Disagree more | Not sure | Strongly agree | Strongly agree |
| --- | --- | --- | --- | --- | --- |
| I perceive that I have gained the income I deserve. | 1 | 2 | 3 | 4 | 5 |
| I perceive that I have gained the social status I deserve. | 1 | 2 | 3 | 4 | 5 |
| I perceive that I have gained the respect and trust of my patients. | 1 | 2 | 3 | 4 | 5 |
| I perceive that I have gained the recognition of my organization. | 1 | 2 | 3 | 4 | 5 |
| I perceive that I have gained the development of my profession. | 1 | 2 | 3 | 4 | 5 |

**Part B Psychological availability scale**

| Below is a description of your workplace and professional status, please select the option that best matches your actual situation (in general) by ticking the appropriate number. | Strongly disagree | Disagree more | Not sure | Strongly agree | Strongly agree |
| --- | --- | --- | --- | --- | --- |
| I am confident in my ability to handle competing demands at work. | 1 | 2 | 3 | 4 | 5 |
| I am confident in my ability to deal with problems that come up at work. | 1 | 2 | 3 | 4 | 5 |
| I am confident in my ability to think clearly at work. | 1 | 2 | 3 | 4 | 5 |
| I am confident in my ability to display the appropriate emotions at work. | 1 | 2 | 3 | 4 | 5 |
| I am confident that I can handle the physical demands at work. | 1 | 2 | 3 | 4 | 5 |

**Part C Future perceived professional benefits scale**

| Below is a description of your workplace and professional status, please select the option that best matches your actual situation (in general) by ticking the appropriate number. | Strongly disagree | Disagree more | Not sure | Strongly agree | Strongly agree |
| --- | --- | --- | --- | --- | --- |
| I perceive that I will gain the income I deserve in the future. | 1 | 2 | 3 | 4 | 5 |
| I perceive that I will gain the social status I deserve in the future. | 1 | 2 | 3 | 4 | 5 |
| I perceive that I will gain the respect and trust of my patients in the future. | 1 | 2 | 3 | 4 | 5 |
| I perceive that I will gain the recognition of my organisation in the future. | 1 | 2 | 3 | 4 | 5 |
| I perceive that I will gain the development of my profession in the future. | 1 | 2 | 3 | 4 | 5 |

**Part D Job engagement scale**

| Below is a description of your workplace and professional status, please select the option that best matches your actual situation (in general) by ticking the appropriate number. | Strongly disagree | Disagree more | Not sure | Strongly agree | Strongly agree |
| --- | --- | --- | --- | --- | --- |
| I really “throw” myself into my job. | 1 | 2 | 3 | 4 | 5 |
| Sometimes I am so into my job that I lose track of time. | 1 | 2 | 3 | 4 | 5 |
| This job is all consuming; I am totally into it. | 1 | 2 | 3 | 4 | 5 |
| My mind often wanders and I think of other things when doing my job. | 1 | 2 | 3 | 4 | 5 |
| I am highly engaged in this job. | 1 | 2 | 3 | 4 | 5 |

**Part E Psychological detachment scale**

| Below is a description of your workplace and professional status, please select the option that best matches your actual situation (in general) by ticking the appropriate number. | Strongly disagree | Disagree more | Not sure | Strongly agree | Strongly agree |
| --- | --- | --- | --- | --- | --- |
| After work, I forget about work. | 1 | 2 | 3 | 4 | 5 |
| After work, I don’t think about work at all. | 1 | 2 | 3 | 4 | 5 |
| After work, I distance myself from my work. | 1 | 2 | 3 | 4 | 5 |
| After work, I get a break from the demands of work. | 1 | 2 | 3 | 4 | 5 |

Please confirm that you have filled them all out, and thank you again for your participation!
